# Enhancing Aircraft Safety through Advanced Engine Health Monitoring with Long Short-Term Memory

**DOI:** 10.3390/s24020518

**Published:** 2024-01-14

**Authors:** Suleyman Yildirim, Zeeshan A. Rana

**Affiliations:** 1 Digital Aviation Research and Technology Centre (DARTeC), Cranfield University, Bedford MK43 0AL, UK; 2 Centre for Aeronautics, Cranfield University, Bedford MK43 0AL, UK

**Keywords:** remaining useful life, predictive maintenance, aircraft health monitoring

## Abstract

Predictive maintenance holds a crucial role in various industries such as the automotive, aviation and factory automation industries when it comes to expensive engine upkeep. Predicting engine maintenance intervals is vital for devising effective business management strategies, enhancing occupational safety and optimising efficiency. To achieve predictive maintenance, engine sensor data are harnessed to assess the wear and tear of engines. In this research, a Long Short-Term Memory (LSTM) architecture was employed to forecast the remaining lifespan of aircraft engines. The LSTM model was evaluated using the NASA Turbofan Engine Corruption Simulation dataset and its performance was benchmarked against alternative methodologies. The results of these applications demonstrated exceptional outcomes, with the LSTM model achieving the highest classification accuracy at 98.916% and the lowest mean average absolute error at 1.284%.

## 1. Introduction

Maintaining an aircraft engine is a complex, time-consuming and expensive process. Direct engine maintenance costs make up approximately 30% of the total maintenance cost of an aircraft [1]. Historically, aircraft engine maintenance was mostly performed at fixed time intervals. However, with the advancement of the aviation industry, it has been realised that this approach is not accurate. Today, engine maintenance is only conducted when needed and efforts are made to reduce the number of fixed-time maintenance events.

In modern aircraft engines, a large number of sensors are installed to assess the condition of the engine. Data from these sensors are evaluated to attempt to predict the engine’s remaining useful life (RUL). The aim of predicting the useful life is to foresee potential damage and malfunctions in the engine before any accidents occur and to carry out preventive maintenance activities. These predictive maintenance activities based on predictions are also known as prognostic maintenance [1].

Maintenance activities for aircraft engines can be divided into two main groups. Maintenance activities that involve the replacement of parts with limited service life due to effects like metal fatigue and microscopic damage are referred to as fixed-time maintenance activities. In fixed-time maintenance, the relevant part is replaced without considering its current condition. Condition-based activities, on the other hand, are preventive primary maintenance processes. They require regular inspection or testing against a specific physical standard to determine whether a device or component can continue to operate. Accurately determining when this inspection and, if necessary, the repair process should be performed plays a crucial role in reducing maintenance costs [1,2].

It is observed that maintenance predictions have been attempted using various methods. Xu et al. attempted to monitor the health of an aircraft engine using data from twenty-one sensors from a hundred different engines [3]. After examining the collected data, all data except the information obtained from seven sensors were eliminated due to insufficient information about the engine’s condition. The remaining data were predicted using a combined system consisting of Dempster–Shafer regression (DSR), a support vector machine (SVM), a recurrent neural network (RNN) and the Mean Squared Error (MSE) was measured as 3.49. Malhotra et al. processed the data of 80 randomly selected engines from the NASA Turbofan Engine Corruption Simulation dataset [4] using encoder–decoder Long Short-Term Memory (LSTM-ED) without any supervision [5]. In the Prognostics and Health Management 2008 Conference Proceedings, the results of the conference’s RUL competition listed the top three performing methods as a hybrid approach based on the similarity-based approach (SBA), an RNN, a Kalman filter and a multi-layer perceptron (MLP) [6].

Zhang et al. predicted the health of the engine using non-linear adaptive predictors on a subset selected from the Commercial Modular Aero-Propulsion System Simulation (C-MAPSS) dataset [7]. Babu et al. processed the C-MAPSS and PHM08 datasets using support vector regression (SVR), an MLP, relevance vector regression (RVR) and a convolutional neural network (CNN) [8]. In this study, they attempted to monitor the health of an engine using data obtained from the 21 sensors of 100 different engines. It was determined that 14 of the sensors they used did not provide useful information about the health of the engine. The assessment was carried out using the data from the remaining 7 sensors. In another study that used Deep Belief Networks (DBNs), two subsets selected from the C-MAPSS dataset were evaluated [9]. The highest classification accuracy in this study was slightly above 90% and achieved with a Multiobjective Deep Belief Network Ensemble (MODBNE).

A recent study conducted by Peng et al. introduced a method for predicting RUL by combining a 1-D CNN with a complete convolutional layer and LSTM [10]. This approach aims to capture both spatial and temporal features in the FD001 and FD003 datasets. Researchers in RUL-related fields have shown considerable interest in CNN applications [11]. The initial utilisation of the CNN methods for predicting RUL was pioneered by Babu et al. [8]. Their findings demonstrated the superior performance of CNNs compared to SVM, MLP and SVR methods.The introduced CNN technique underwent testing and assessment using the C-MAPSS dataset, yielding an Root Mean Square Error (RMSE) of 18.45. Zhao et al. employed a two-channel hybrid model to predict the RUL of engines and this model outperformed traditional prediction models [12].

Kong et al. [13] introduced a predictive approach for estimating the RUL. This method involved employing an adaptive time series feature window in conjunction with a multi-step forward technique. Zhuang et al. [14] utilised a confrontational online regression strategy from multiple sources to predict RUL, specifically addressing online unknown conditions. Yao et al. [15] presented a robust neural network while Yan et al. [16] introduced an algorithm for diagnosing faults that considers the complete life cycle, spanning from normal functioning to eventual failure. This life cycle was segmented into multiple degradation stages and the prediction of these stages was accomplished using a combination of a Hidden Markov Model and an SVM enhanced through particle swarm optimisation. Deep learning with its intricate multilevel internal architecture and the benefits of relearning training approaches can address the limitations associated with traditional manual feature extraction. An aspect of RUL prediction involves temporal correlation. Despite this, the conventional methods mentioned earlier lack the capability to harness the temporal correlation characteristics present in time series data, hindering their ability to make more precise and accurate predictions of RUL. To address this challenge, Han et al. [17] implemented an approach that incorporated stacked auto-encoders and RNNs to forecast the RUL. Liu et al. [18] introduced a method for predicting the RUL of lithium-ion battery capacity using the Gated Recurrent Unit (GRU). Guo et al. [19] suggested employing LSTM for forecasting time series associated with mechanical breakdown.

Ensarioglu et al. [20] offered a novel strategy for the prediction of RUL involving the creation of features based on differences, labelling through change-point detection and piecewise linear techniques and the utilisation of a hybrid 1D-CNN-LSTM neural network. Li et al. [21] presented a data-driven approach employing a CNN with a time window strategy for sample preparation, aiming to improve feature extraction. In a different approach, Yu et al. [22] suggested a two-step method which included a bidirectional RNN autoencoder followed by similarity-based curve matching for RUL estimation. The autoencoder transformed high-dimensional sensor data into low-dimensional representation embeddings to facilitate subsequent similarity matching. Peng et al. [23] designed a dual-channel LSTM model that dynamically selects time features and conducts initial processing on time-feature values. This involves using LSTM to extract time features and first-order time-feature information. Wang et al. [24] proposed a bespoke multi-scale LSTM for addressing three distinct degradation stages of aircraft engines: the constant stage, transition stage and linear degradation stage. In dealing with different data distributions during training and testing, Lyu et al. [25] employed LSTM for sensor data feature extraction and implemented multi-representation domain adaptation methods to provide effective feature alignment between source and target domains. Deng et al. [26] introduced an innovative multi-scale dilated CNN incorporating a new multi-scale dilation convolution fusion unit to enhance the receptive field and operational efficiency when predicting the RUL of critical components in mechanical systems.

When the current literature is examined, it is observed that a vast majority of recent studies are data-driven whilst model-based studies have lost their relevance. Data-driven studies can be categorised into two main groups. In the first group of studies, attempts are made to determine the RUL. In such studies, historical data obtained from the engine are treated as a time series and the problem is transformed into a regression problem [3,5,8]. In the second group of studies, the condition of the engine is categorised into classes such as healthy, slightly damaged and heavily damaged, reducing the problem to a classification problem [7,9,27]. In this study, the condition of the engine has been categorised into two groups based on the RUL as “healthy” and “needs maintenance”. The objective of this study is to perform predictive maintenance assessment in aircraft health monitoring using an LSTM-based solution. The success of deep learning is evaluated by comparing it with regression and artificial neural network models. The dataset used in this study is the freely distributed C-MAPSS dataset provided by NASA.

## 2. Methodology

The dataset commonly used in the literature for predictive maintenance in aircraft health systems is the NASA Turbofan Engine Corruption Simulation dataset [4]. The dataset was created by NASA engineers using commercial simulation software called C-MAPSS version 2. The software simulates a turbofan engine capable of generating 90,000 pounds of thrust at altitudes of 0–40,000 feet and speeds of 0–0.9 Mach with ambient temperatures ranging from −60 to 103 degrees Fahrenheit. The software also includes various regulators and limiters that prevent the engine from operating outside the manufacturer-specified operating range.

The engine was operated in conjunction with the control system during dataset creation. The simulation was halted when the health index of the engine dropped to zero and the acquired sensor data were recorded as a time series. The health index of the engine is defined to be one under ideal conditions for each component of the engine and it becomes zero when the specified operating conditions are exceeded. Training data were continued until the health index of the engine reached zero whilst test and validation data were terminated before the engine malfunction to measure RUL. The difference between the cycle in which the engine health index drops to zero and the cycle in which it is currently present provides the RUL value of the engine. The dataset includes four subsets prepared for different operating conditions and scenarios. In the subset used in this study, data from 21 sensors were collected from 100 engines. A total of 20,631 cycles were recorded for training data and 13,096 cycles for testing. The data are labelled with two categories: “needs maintenance” or “healthy”. Engines with an RUL value below 150 are considered to require maintenance.

The LSTM was trained on the Hypercomputing Integrated Layer for Digital Aviation High-performance Computing (HPC) at Cranfield University. This computational system was fitted with 4 ${\mathrm{NVIDIA}}^{®}$ A100 80 GB GPUs, 112 ${\mathrm{Intel}}^{®}$ Xeon Gold 6258R CPU, 330 Tb of storage capacity and 377 GB of DDR4-2933 RAM [28]. These dedicated computational resources were specifically allocated for this study. The development environment was established as a Python 3.7.0 environment with careful consideration of the installation of specific versions of essential packages and dependencies. The environment was equipped with TensorFlow 2.3.0, Keras 2.3.1, PyTorch 1.7.1+cu101, scikit-learn 0.24.0, pandas 1.1.5 and NumPy 1.21.6, each contributing to the execution of diverse computational tasks. Furthermore, the system was optimised for GPU acceleration as demonstrated by the inclusion of CUDA-related packages, cudatoolkit 10.1.243 and cudnn 7.6.5.

### Long Short-Term Memory

**Figure 1:**
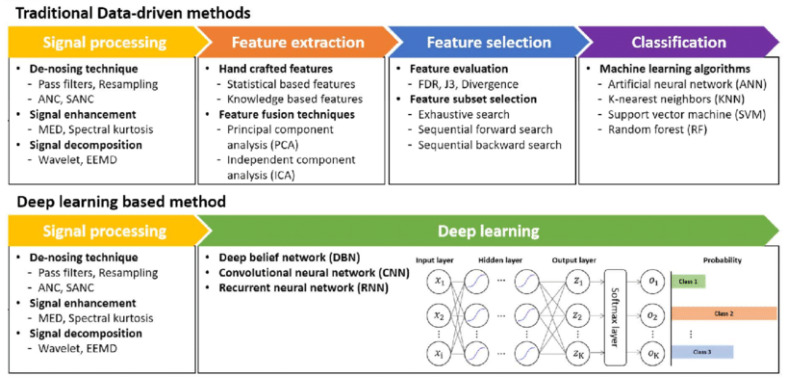
Traditional data-driven vs. deep-learning-based methods [30].

Deep learning methods have gained popularity in recent years. Initially applied more in image processing, these methods have gradually been successfully utilised in health monitoring over time [29]. Processing meaningful features from raw data is one of the crucial processing steps in a learning problem. The most significant advantage of deep learning methods is their ability to work directly on raw data without the need for predefined features during the learning phase. However, this advantage can also turn into a disadvantage as it brings along a high computational cost. Figure 1 illustrates a comparison between traditional data-driven approaches and deep-learning-based methods.

Health monitoring data are predominantly in time series or time-dependent functions. Deep learning methods capable of processing such data are recurrent neural networks. The LSTM has been utilised in this study to predict the health of the aircraft engine as one of the recurrent deep learning methods. To facilitate the comprehension of the LSTM architecture, the initial focus will be on the Gated Recurrent Unit as a simplified explanatory structure followed by a comprehensive exploration of the LSTM. The GRU is essentially a type of RNN architecture that is effective in capturing past information to explain current information and predict future information. RNNs produce successful results in solving problems defined as time series or time-dependent functions [31].

**Figure 2:**
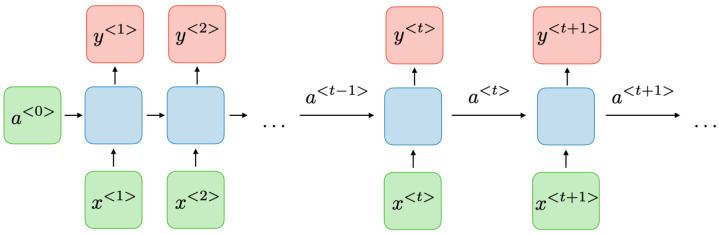
Recurrent neural network architecture [32].

**Figure 3:**
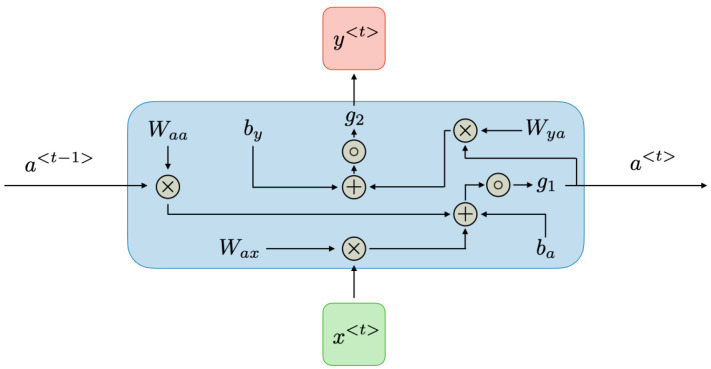
Recurrent neural network diagram [32].

Figure 2 and Figure 3 illustrate the architecture and the block diagram of an RNN respectively. $i^{\langle T_{y}\rangle }$ represents the input value fed into the x layer at the corresponding time. The weight values are expressed by *W* and the bias values are represented by b . ${\hat{y}}^{\langle T_{y}\rangle }$ is the prediction calculated at the output of each neuron at the respective time and using the predictions calculated at all time steps, the loss function $L^{\langle T_{y}\rangle }$ is computed.

At each time step *t*, the activation $a^{<t>}$ and the output $y^{<t>}$ can be described as follows:

(1) $$a^{<t>}=g_{1}\left(W_{aa}a^{<t-1>}+W_{ax}x^{<t>}+b_{a}\right)$$

(2) $$y^{<t>}=g_{2}\left(W_{ya}a^{<t>}+b_{y}\right)$$

where $W_{ax},W_{aa},W_{ya},b_{a},\;b_{y}$ are coefficients shared across time and $g_{1}$ , $g_{2}$ represent activation functions.

The overall loss function is estimated using the results from all time steps. The loss function can be calculated as shown in Equation (3).

(3) $$L\left(\hat{y},y\right)=\sum_{t=1}^{T_{y}}L\left({\hat{y}}^{<t>},y^{<t>}\right)$$

Backpropagation is performed at every time step to facilitate the learning process. At time step *T*, the derivative of the loss L with respect to the weight matrix *W* can be formulated as follows:

(4) $${\left.\frac{\partial L^{\left(T\right)}}{\partial W}=\sum_{t=1}^{T}\;\frac{\partial L^{\left(T\right)}}{\partial W}\right|}_{\left(t\right)}$$

**Figure 4:**
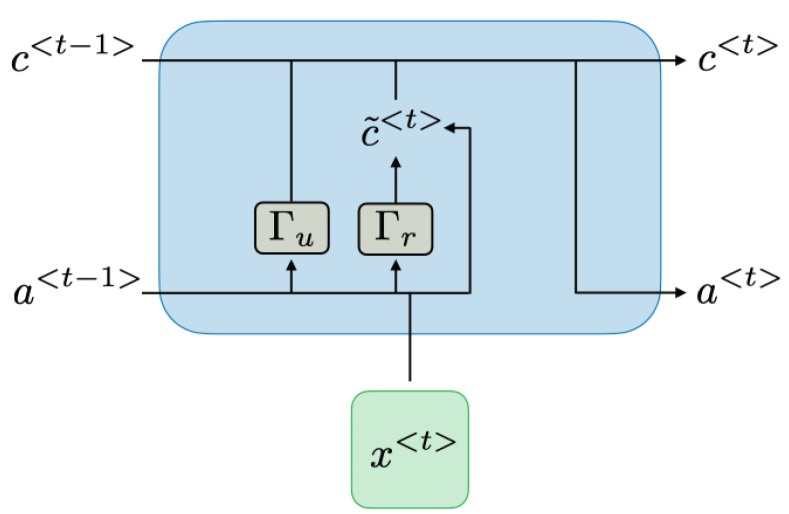
Gated Recurrent Unit diagram [32].

RNNs can be classified based on the activation functions in their x layers and the manner in which temporal processes are handled. The connections and activation processes within these internal units directly impact the memory characteristics of the model. Processes such as update and reset are performed in these units. GRUs are among the simplest basic units that can be used [31]. Figure 4 illustrates a GRU block diagram.

The value denoted as $c^{\langle t\rangle }$ is equal to $a^{\langle t\rangle }$ and represents the memory. The ${\tilde{c}}^{\left(t\right)}$ value symbolises the candidate cell state for the memory. The hyperbolic tangent is used as the activation function. The candidate cell state can be calculated using Equation (5):

(5) $$\tanh(W_{c}\left[\Gamma_{r}\times a^{<t-1>},x^{<t>}\right]+b_{c})$$

Additionally, update and reset gate values are calculated in GRUs. The sigmoid function is used as the activation function for these values. While the update gate determines how much of the previous memory content should be retained and how much of the new candidate values should be added to the memory, the reset gate determines how much of the previous state should be forgotten and how much of the new input should be used to compute the new candidate state. The update gate $\Gamma_{u}$ and reset gate $\Gamma_{r}$ are computed as follows:

(6) $$\Gamma_{u}=\sigma \left(W_{u}\left[{\tilde{c}}^{\left(t-1\right)},x^{\left(t\right)}\right]+b_{u}\right)$$

(7) $$\Gamma_{r}=\sigma \left(W_{r}\left[{\tilde{c}}^{\left(t-1\right)},x^{\left(t\right)}\right]+b_{r}\right)$$

Using the value of $\Gamma_{u}$ , the $c^{\left(t\right)}$ value can be calculated for the new time step even if there is no new update with the help of previous information, as shown in Equation (8).

(8) $$c^{\langle t\rangle }=\;\Gamma_{u}\times {\tilde{c}}^{\langle t\rangle }+\left(1-\Gamma_{u}\right)\times {\tilde{c}}^{\langle t-1\rangle }$$

**Figure 5:**
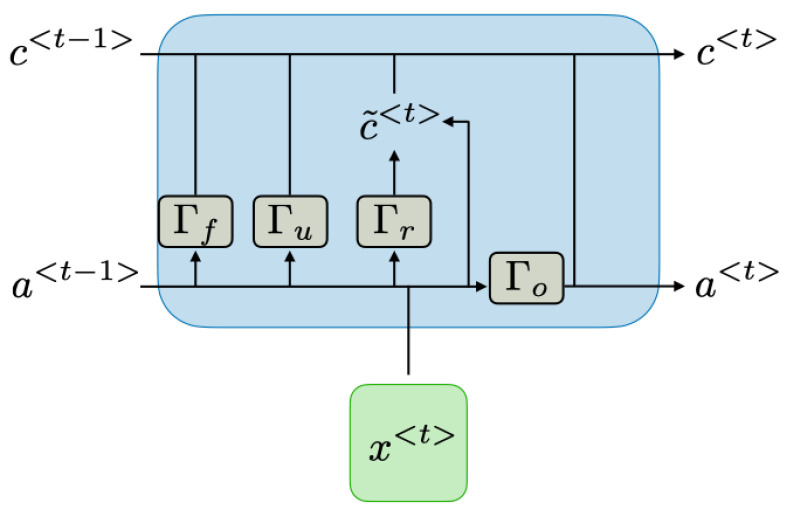
LSTM diagram [32].

LSTM is a specialised version of the GRU structure. Features that comprehend past and future information are recurrently carried in the LSTM structure. A simple LSTM structure is displayed in Figure 5. Activation functions are employed at three distinct points in LSTM. While the sigmoid function is always employed in the forget gate, the hyperbolic tangent function is commonly used in the input and output layers. The most significant difference of this structure from the GRU is that the reset gate in the LSTM structure is specialised to obtain forget gate $\Gamma_{f}$ and output gate $\Gamma_{o}$ with Equations (9) and (10). The forget gate allows reducing the weights of information transferred from the past that may not be necessary. With the combination of the update gate and forget gate, a more effective output is generated [33].

(9) $$\Gamma_{f}=\sigma \left(W_{f}\left[a^{\langle t-1\rangle },x^{\langle t\rangle }\right]+b_{f}\right)$$

(10) $$\Gamma_{o}=\sigma \left(W_{o}\left[a^{\langle t-1\rangle },x^{\langle t\rangle }\right]+b_{o}\right)$$

Therefore, the new $a^{\langle t\rangle }$ value can be calculated as given in Equation (11).

(11) $$c^{\langle t\rangle }=\Gamma_{u}\times {\tilde{c}}^{\langle t\rangle }+\left(\Gamma_{f}\right)\times {\tilde{c}}^{\langle t-1\rangle }$$

(12) $$\;\;\;c^{\langle t\rangle }*\Gamma_{o}=a^{\langle t\rangle }$$

**Figure 6:**
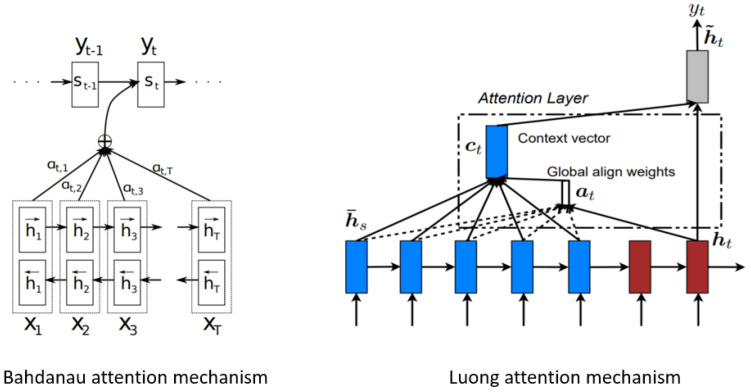
Conventional attention mechanisms [35].

Attention mechanisms are a fundamental concept that allows LSTM to selectively focus on specific parts of input sequences when making predictions as seen in Figure 6. This mechanism enables the LSTM to weigh the importance of different elements dynamically, enhancing its ability to capture relevant information. In the context of LSTM, attention mechanisms are applied to address challenges related to handling sequential data with long-range dependencies. LSTMs suffer from difficulties in capturing contextual information across extended sequences. By integrating attention mechanisms, LSTMs learn to assign varying degrees of significance to different parts of the input sequence. This allows the LSTM to focus more on relevant information and improves its performance on tasks such as time-series analysis and sequential prediction problems. This combination of attention mechanisms with LSTMs has proven effective in various applications where understanding contextual relationships in sequential data is crucial for accurate predictions [34].

In predicting RUL in the C-MAPSS dataset, the attention layer within the LSTM architecture allows the LSTM to dynamically weigh the significance of individual sensor readings across the entire sequence of engine health data. This adaptive focus ensures that the LSTM places greater emphasis on time steps that are more indicative of impending system degradation. By attending to relevant temporal patterns, the LSTM with attention better captures subtle nuances in the data, leading to improved RUL predictions. The advantages lie in its ability to handle sequences of varying lengths effectively and discern patterns critical for predictive accuracy, making it particularly well suited for time-series forecasting tasks like RUL prediction in the C-MAPSS dataset.

Rather than implementing the traditional attention mechanism within an encoder–decoder architecture, this study explores the potential application of an attention mechanism operating independently from an encoder–decoder architecture. When integrating attention mechanisms with an LSTM architecture devoid of an encoder–decoder structure, the approach involves utilising the LSTM’s hidden states directly for computing attention scores and generating context vectors. The sequence processing begins with an input sequence of length *T* where each time step is represented by features $X_{t}$ and the LSTM processes this sequence to derive hidden states $H_{t}$ for each time step. Key vectors $K_{t}$ and value vectors $V_{t}$ are computed directly from the LSTM hidden states through learnable weight matrices $W_{k}$ and $W_{v}$ . The query vector *Q* for the current step is derived from the current LSTM hidden state using another learnable weight matrix $W_{q}$ . Attention scores $e_{t}$ are calculated using a similarity measure such as the dot product between the query vector and key vectors. The Adam algorithm is then applied to obtain attention weights $a_{t}$ . The context vector *C* is computed as the weighted sum of value vectors using these attention weights. This context vector is concatenated with the LSTM hidden state at the current step, forming an attention output. This concatenated vector can be further processed through additional layers, leading to the generation of the final output. The entire process is repeated for each step, allowing the LSTM to dynamically focus on different parts of the input sequence based on the evolving context. This approach proves particularly effective in tasks requiring the modelling of intricate temporal relationships such as time-series prediction or sequence-to-sequence tasks without a distinct encoder–decoder architecture.

(13) $$e_{t}=\mathrm{Dot}\left(Q,K_{t}\right)$$

(14) $$a_{t}=\mathrm{Adam}\left(e_{t}\right)$$

(15) $$C=\sum_{t=1}^{T}a_{t}\cdot V_{t}$$

Hyperparameter tuning in LSTM is essential to optimise the LSTM configuration, ensuring it captures temporal dependencies effectively, prevents overfitting and adapts to the specific characteristics of the dataset. It enhances LSTM performance, leading to more accurate predictions and improved generalisation of new data. Therefore, hyperparameter tuning is a critical process where hyperparameters are systematically adjusted to optimise LSTM performance [36]. Instead of manually adjusting these settings, autotuning employs optimisation algorithms to systematically explore the hyperparameter space and discover the optimal configuration as seen in Algorithm 1. Autotuning in LSTM involves the systematic optimisation of configuration settings that are not learned during the training process but are essential for defining the LSTM’s architecture and behaviour. This automated approach helps save time and computational resources while uncovering fine combinations that lead to improved LSTM performance. By adjusting hyperparameters such as the learning rate, number of LSTM units, dropout rates and activation functions, the LSTM’s ability to capture intricate temporal patterns and generalise to new data is significantly improved. This process helps prevent issues like overfitting or underfitting, ensuring that the LSTM adapts optimally to the unique characteristics of a given dataset. Moreover, hyperparameter tuning allows for the exploration of the vast search space, identifying configurations that lead to more efficient convergence during training and better overall predictive accuracy.
**Algorithm** **1** Creating Tuner Object.
1:**Initialise** tuner2:    **Input:**3:       *build_model*—Function to define the model architecture4:       *objective = ‘mse’*—Optimisation objective5:       *max_trials = 5*—Maximum number of hyperparameter combinations to try6:    *executions_per_trial = 3*—Number of times to train the same architecture with different initialisation7:**End Initialise**


The provided pseudocode above utilises the initiation process for a RandomSearch Tuner. The algorithm initialises the tuner at the outset, specifying essential parameters. The build_model input signifies a function that defines the architecture of the LSTM subject to tuning while the objective parameter sets the optimisation goal. The max_trials parameter dictates the maximum number of hyperparameter combinations the tuner will explore and executions_per_trial determines how many times the same architecture will be trained with different initialisations. The “End Initialise” section marks the completion of the initialisation process. This pseudocode provides a concise representation of the key components involved in configuring a RandomSearch Tuner for hyperparameter optimisation.

Algorithm 2 executes a hyperparameter tuning process for LSTM. The tuner.search() function initiates the hyperparameter search, exploring different combinations of hyperparameter values to find the optimal configuration for the LSTM. The $x=X_{train}$ and $y=Y_{train}$ parameters specify the training data and corresponding labels used during the search. The model is trained for 20 epochs with a batch size of 128. The validation of the model is performed on a separate dataset— $X_{test}$ and $Y_{test}$ —to assess its generalisation performance. The overall goal of this hyperparameter tuning process is to identify the set of hyperparameters that maximises the LSTM’s effectiveness. This search aims to discover the configuration that yields the best validation performance among the explored options.
**Algorithm** **2** Hyperparameter Tuning with Algorithmic Search.      
**Input:** Training data 
$X_{\mathrm{train}}$
, 
$Y_{\mathrm{train}}$
, validation data 
$X_{\mathrm{test}}$
, 
$Y_{\mathrm{test}}$

  2:  **Output:** Optimal hyperparameters for LSTM
       Initialise hyperparameter search space and define the search algorithm
  4.  **while** not reached maximum number of iterations **do**
           Sample a set of hyperparameters from the search space
  6:      Build an LSTM with the sampled hyperparameters
           Train the model on 
$X_{\mathrm{train}}$
 and 
$Y_{\mathrm{train}}$
 for a fixed number of epochs using batch size 128
  8:      Evaluate the model on the validation set 
$\left(X_{\mathrm{test}},Y_{\mathrm{test}}\right)$

           Update the search algorithm’s internal state based on the performance of the model
10:      **if** current model’s performance is better than the best so far **then**
               Update the best hyperparameters
12:      **end if**
       **end while**
14:  **Return:** Best hyperparameters found during the search


The application of an attention mechanism without an encoder–decoder architecture coupled with the autotuning LSTM hyperparameters constitutes a substantial advancement particularly demonstrated in the enhanced prediction of RUL on the C-MAPSS dataset. The exclusion of an explicit encoder–decoder structure signifies a departure from traditional attention mechanisms wherein direct utilisation of LSTM hidden states for attention computation enables a more streamlined and context-aware information processing. The incorporation of autotuning techniques further refines the LSTM’s predictive capacity by systematically optimising hyperparameters, allowing the LSTM to adapt dynamically to the dataset characteristics. This approach not only underscores the significance of attention mechanisms in sequence modelling but also highlights the efficacy of autotuning in fine-tuning LSTM architectures for improved prognostic accuracy in the predictive maintenance domain and reliability engineering.

**Figure 7:**
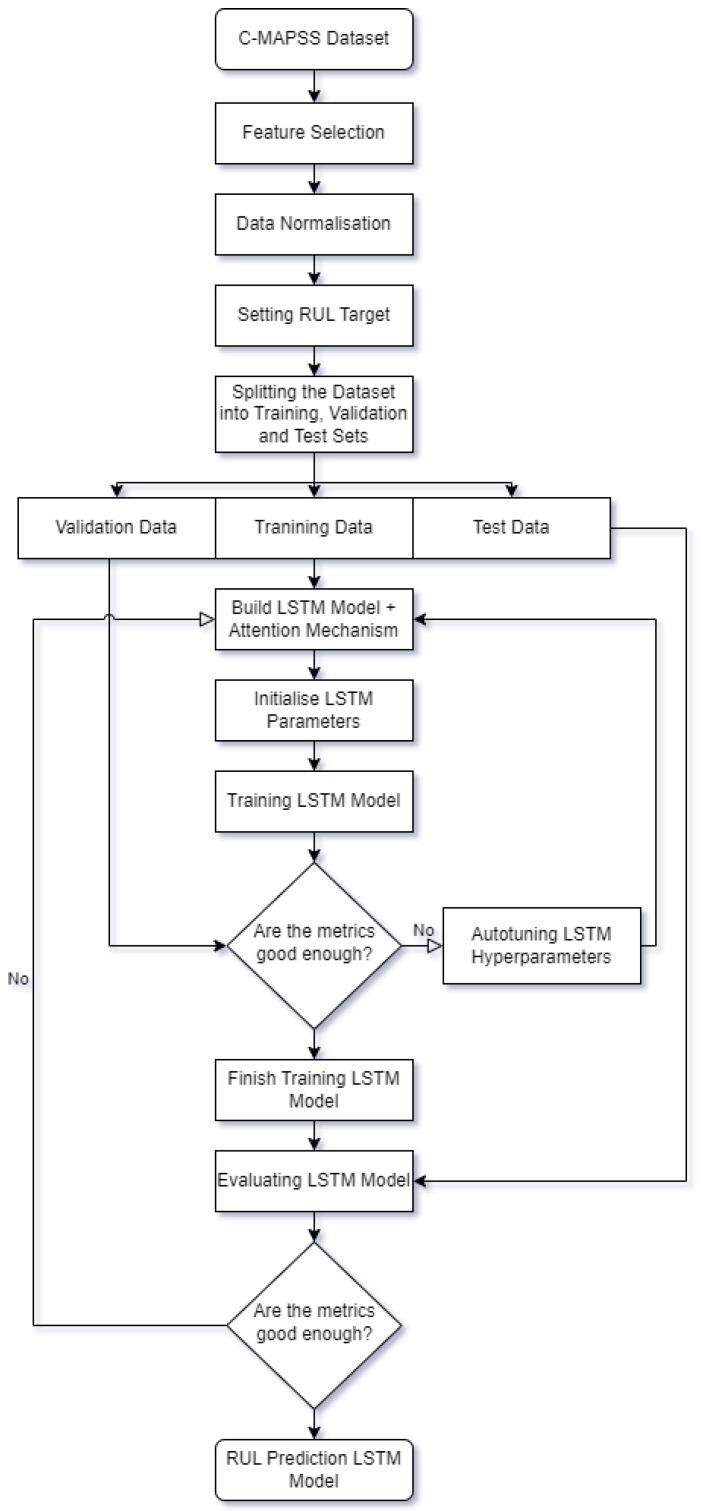
Advanced engine health monitoring flowchart.

The advanced engine health monitoring employed in this study is outlined in Figure 7, illustrating the sequential steps undertaken for prognostics using the C-MAPSS dataset. To begin, the dataset is split into three subsets, designated for validation, training and testing purposes. Feature selection and data normalisation are then applied. Following these preprocessing steps, LSTM hyperparameters are initialised and an LSTM model incorporating an attention mechanism is constructed. The initial training of the LSTM model takes place using the training data. In the event that the performance metrics do not meet predefined criteria, an autotuning process is initiated to optimise the LSTM hyperparameters, prompting the reinitialisation and reconstruction of the LSTM model for additional training cycles. Conversely, if the metrics demonstrate satisfactory results, the training concludes and the model undergoes evaluation using the test set. Should the evaluation metrics meet the desired threshold, the training process is stopped. Contrarily, if the metrics do not meet predefined criteria, the training process recommences, iterating until the desired performance is achieved. This approach ensures a systematic and iterative refinement of the LSTM model for optimal RUL prediction capabilities.

## 3. Results

### 3.1. C-MAPSS Dataset

**Figure 8:**
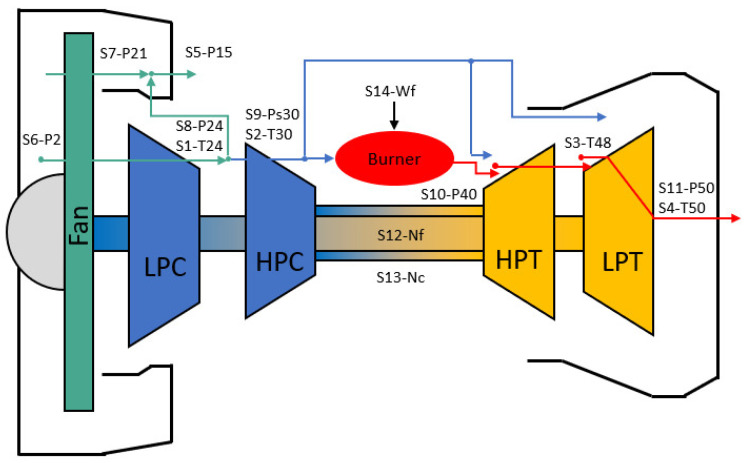
C-MAPSS model representation [37].

To generate realistic run-to-failure trajectories, it is crucial to have a suitable system model that can accommodate variations in the health of sub-systems and simulate sensor measurements. The C-MAPSS dynamical model is a highly accurate computer model designed for simulating a large commercial turbofan engine realistically. Figure 8 depicts an illustrative diagram of the engine, along with the associated station numbers as specified in the C-MAPSS model documentation [37]. Alongside the thermodynamic model for the engine, the dataset contains an atmospheric model that can function across altitudes ranging from sea level to 40,000 feet, Mach numbers from 0 to 0.90 and sea-level temperatures between −60 and 103 degrees Fahrenheit. Additionally, there is a power-management system integrated, enabling the engine to operate across a broad spectrum of thrust levels across all flight conditions.

The C-MAPSS system model takes the shape of an interconnected set of nonlinear equations. The inputs to this system model are categorised into two parts: scenario-description operating conditions denoted as *w* and hidden model health parameters represented as θ . The outputs produced by the system model consist of approximations for physically measurable properties denoted as $x_{s}$ and unobservable properties $x_{v}$ that are not part of the condition monitoring signals. This nonlinear system model is identified in Equation (16). The unobservable model health parameters serve as adjusters within the model and belong to the category known as quality parameters. These parameters encompass elements such as component efficiencies, flow, input and output scalars and additional modifying factors. They are instrumental in emulating the deteriorated behaviour of the system. Specifically, all the rotating sub-components of the engine—the fan, low-pressure compressor (LPC), high-pressure compressor (HPC), low-pressure turbine (LPT) and high-pressure turbine (HPT)—can experience degradation in both flow and efficiency.

(16) $$\left[x_{s}^{\left(t\right)},x_{v}^{\left(t\right)}\right]=F\left(w^{\left(t\right)},\theta^{\left(t\right)}\right)$$

Table 1 provides an overview of the sensor data associated with turbofan engine. Each row in the table corresponds to a specific sensor with columns detailing the sensor number, a concise description of its function and the respective units of measurement. The data encompass a range of parameters critical for monitoring the health and performance of turbofan engines, including temperatures at various points in the engine (such as the fan inlet, LPC outlet, HPC outlet and LPT outlet), pressures (fan inlet and bypass duct) and speed related metrics (physical fan speed and physical core speed). The table also captures information on engine pressure ratios, fuel–air ratios and various airflow parameters.

### 3.2. Experimental Analysis

This study proposes an aircraft health monitoring solution based on the prediction of the RUL for aircraft engines. It was emphasised that the LSTM which is a type of RNN, can provide a suitable solution to the problem defined above. The obtained results have been compared with traditional methods. The sensor data from 100 engines selected from the C-MAPSS dataset curated by NASA were employed in the training process. A total of 20,631 randomly selected instances of data were allocated for training while 13,096 instances were reserved for testing. The test data were labelled to transform the problem into a classification task. The labels indicate that engines with RUL values less than 150 “needs maintenance” whilst others are considered “healthy”. A three-layer LSTM structure was established in the model and a dropout layer was implemented. The first two LSTM layers consist of 100 LSTM units each and the final layer comprises 75 units. A dropout rate of 0.5 has been selected for the dropout operation. Binary cross-entropy has been utilised for the loss function. Binary cross-entropy loss measures the performance of a classification model with output probability values between 0 and 1. The cross-entropy loss increases as the predicted probability diverges from the actual label. The binary cross-entropy loss is calculated as in Equation (17). In the given formula, *N* is the number of classes, $y_{i}$ is a binary indicator representing whether class *i* is the correct class and $p_{i}$ is the predicted probability assigned to class *i* by the model.

(17) $$H_{i}=-\sum_{i=1}^{N}y_{i}\cdot \log\left(p_{i}\right)$$

The Adam algorithm initialises two moving averages *m* and *v* representing the first moment estimate (mean) and the second moment estimate (uncentred variance). Each iteration involves calculating the gradient of the LSTM’s loss with respect to each parameter through backpropagation. The first and second moment estimates are updated using decay rates $\beta_{1}$ , $\beta_{2}$ and bias correction is applied to account for initialisation biases. The bias-corrected moment estimates guide the update of model parameters, confirming an adaptive learning rate based on the historical gradients. This adaptive learning rate facilitates efficient convergence and parameter adjustments, making the Adam algorithm particularly effective in navigating the intricate patterns of engine degradation dynamics in RUL prediction. The formulae for these parameters are shown in Equations (18)–(22).

The first moment estimate *m* is updated using the current gradient $g_{t}$ and the decay rate $\beta_{1}$ :

(18) $$m_{t}=\beta_{1}\cdot m_{t-1}+\left(1-\beta_{1}\right)\cdot g_{t}$$

The second moment estimate *v* is updated similarly with the decay rate $\beta_{2}$ :

(19) $$v_{t}=\beta_{2}\cdot v_{t-1}+\left(1-\beta_{2}\right)\cdot {\left(g_{t}\right)}^{2}$$

To account for the initialisation bias of *m* and *v* at the early training steps, bias-corrected estimates ${\hat{m}}_{t}$ and ${\hat{v}}_{t}$ are calculated:

(20) $$\begin{array}{cc} \; & {\hat{m}}_{t}=\frac{m_{t}}{1-\beta_{1}^{t}} \end{array}$$

(21) $$\begin{array}{cc} \; & {\hat{\vartheta }}_{t}=\frac{v_{t}}{1-\beta_{2}^{t}} \end{array}$$

The parameters are updated using the bias-corrected moment estimates:

(22) $$\theta_{t}=\theta_{t-1}-\frac{\mathrm{learning}\;\mathrm{rate}\cdot {\hat{m}}_{t}}{\sqrt{{\hat{v}}_{t}}+\epsilon }$$

The designed model has 183,676 parameters and the optimal values for these parameters have been determined using the Adam algorithm. Its adaptive learning rate mechanism allows the Adam algorithm to adjust the learning rates for individual parameters based on historical gradients which is advantageous when dealing with the diverse scales and behaviours of LSTM parameters. LSTMs often encounter challenges such as vanishing or exploding gradients and the Adam algorithm’s ability to handle sparse gradients is particularly beneficial in such scenarios. The inclusion of a momentum term in the Adam algorithm helps to accelerate the optimisation process, providing smoother convergence and efficient navigation through flat regions in the loss landscape. The Adam algorithm reduces the need for manual tuning of learning rates and its default hyperparameters are known to work well across a variety of problems. While the choice of optimiser can depend on specific problem characteristics, the Adam algorithm’s versatility and solid performance make it a popular and effective option for training LSTMs [38]. The first layer is an LSTM layer with 100 units. This layer has 50,400 parameters which include weights and biases. The second layer is another LSTM layer with the same architecture as the first, featuring 100 units and a total of 80,400 parameters. The increased number of parameters in this layer allows for the extraction of more complex temporal patterns in the data. The third layer is yet another LSTM layer but with a reduced number of units of 75. This layer contributes 52,800 parameters. The choice of reducing the number of units is to extract more compact features and to manage computational complexity. The fourth layer is a dropout layer with 75 units. The dropout layer is used to prevent overfitting by randomly deactivating half of the units during training. The fifth and final layer is a dense layer with a single unit. This layer has 76 parameters which include weights and biases. The combination of these layers, each serving a specific purpose, forms the architecture of the LSTM network.

The accuracy, precision, recall and F1-score metrics have been calculated to evaluate the obtained results. The importance of the F1-score lies in its ability to provide a balanced assessment of the LSTM’s performance [39]. Where the identification of impending failures is crucial, the F1-score addresses the challenge of class imbalance by simultaneously considering precision and recall. As false positives and false negatives can have varying degrees of impact on decision-making in maintenance strategies, the F1-score serves as a holistic metric that captures the trade-off between minimising misclassifications and maximising the capture of true positive instances. Its quantifiable nature and the interpretability it offers make the F1-score an important metric in guiding the development and refinement of LSTM for accurate and reliable RUL predictions for the complex and dynamic C-MAPSS dataset. The formulae for these metrics are presented in Equations (23)–(26). In these equations, TP represents the number of examples correctly identified as “needs maintenance”, FP denotes the number of examples incorrectly identified as “needs maintenance”, TN signifies the number of examples correctly identified as “healthy” and FN indicates the number of examples incorrectly identified as “healthy”. The obtained results are summarised in Table 2. The MAE between the RUL values and the predictions has been calculated to assess the regression performance of LSTM.

(23) $$\mathrm{Accuracy}=\frac{\mathrm{True}\;\mathrm{Positives}+\mathrm{True}\;\mathrm{Negatives}}{\mathrm{Total}\;\mathrm{Samples}}$$

(24) $$\mathrm{Precision}=\frac{\mathrm{True}\;\mathrm{Positives}}{\mathrm{True}\;\mathrm{Positives}+\mathrm{False}\;\mathrm{Positives}}$$

(25) $$\mathrm{Recall}=\frac{\mathrm{True}\;\mathrm{Positives}}{\mathrm{True}\;\mathrm{Positives}+\mathrm{False}\;\mathrm{Negatives}}$$

(26) $$F1-\mathrm{score}=\frac{2\times \mathrm{Precision}\times \mathrm{Recall}}{\mathrm{Precision}+\mathrm{Recall}}$$

(27) $$\mathrm{MAE}=\frac{1}{N}\sum_{i=1}^{N}\left|y_{i}-{\hat{y}}_{i}\right|$$

The same dataset has been utilised with different learning algorithms and the obtained results are used for comparison to assess the success of the proposed solution. The RUL value employed for classifying the data forms a time series, allowing the problem to be initially addressed as a regression problem. Afterwards, classification can be performed based on the predicted RUL values. Linear Regression, Support Vector Regression, K-Nearest Neighbours Regression, Gaussian Processes Regression and Random Forest Regression were used for this comparison.

(28) $$\mathrm{MSE}=\frac{1}{N}\sum_{i=1}^{N}{\left(y_{i}-{\hat{y}}_{i}\right)}^{2}$$

(29) $$\mathrm{RAE}=\frac{\sum_{i=1}^{N}\left|y_{i}-{\hat{y}}_{i}\right|}{\sum_{i=1}^{N}\left|y_{i}-\tilde{y}\right|}$$

(30) $$\mathrm{RSE}=\frac{\sum_{i=1}^{N}{\left(y_{i}-{\hat{y}}_{i}\right)}^{2}}{\sum_{i=1}^{N}{\left(y_{i}-\tilde{y}\right)}^{2}}$$

Various evaluation criteria such as Mean Absolute Error (MAE), Mean Squared Error (MSE), Relative Absolute Error (RAE) and Relative Squared Error (RSE) have been applied to these methods. The formulae are shown in Equations (27)–(30). *N* represents the number of samples, $y_{i}$ is the actual value, ${\hat{y}}_{i}$ is the predicted value and $\tilde{y}$ is the mean of the actual values.

It can be observed from Table 3 that the performance of methods other than Gaussian Processes Regression and Random Forest Regression is quite low. A more detailed comparison of the performances of Gaussian Processes Regression and Random Forest Regression methods which produce relatively better results with LSTM is presented in Table 4.

**Figure 9:**
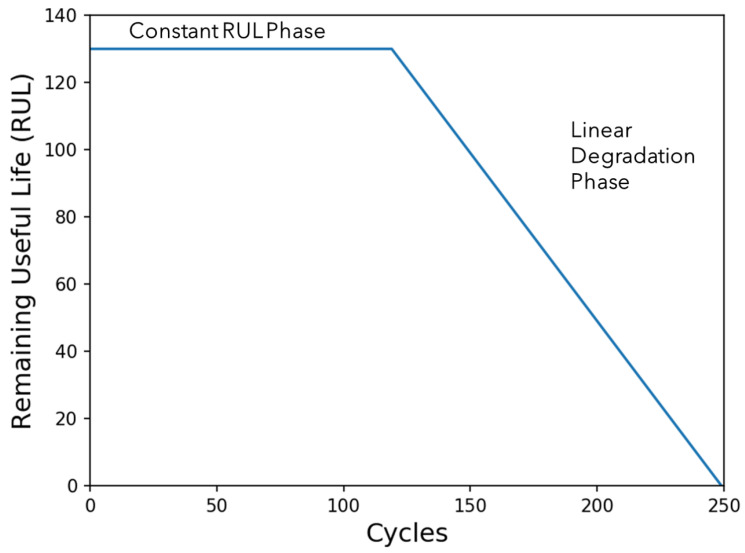
Remaining useful life function.

The target function can be seen in Figure 9 and is designed as a piece-wise function to account for the inherent complexities in the degradation patterns of engines. It has been established that the deterioration of an engine cannot manifest visibly until a certain operational threshold has been surpassed, marking the onset of initial failure. To address this subtle behaviour, the RUL target function is strategically formulated to reflect the gradual degradation process. This modelling approach aligns with the understanding that the RUL for each trajectory within the dataset is reasonably limited to within the range of 125 to 250 cycles, acknowledging that the predictive accuracy of the model should be most robust within this specific temporal scope as engines with an RUL value below 150 are considered to require maintenance. By adopting a piece-wise function, the predictive model aims to capture the distinct phases of engine degradation, facilitating more accurate and context-aware predictions of the RUL across diverse trajectories in the C-MAPSS dataset.

**Figure 10:**
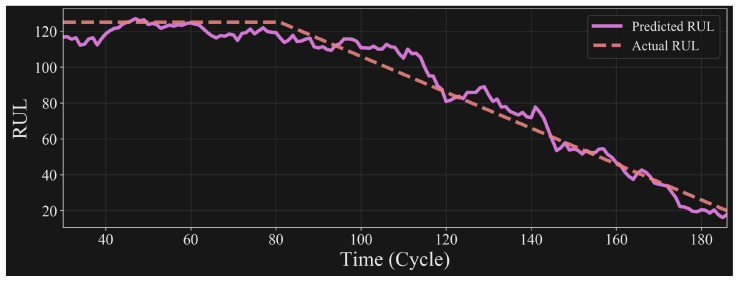
Prediction results.

The results of the LSTM on the test set are visually represented in Figure 10 where the prediction curve corresponds to the LSTM-generated predictions and the actual RUL curve denotes the real RUL values as per FD001, FD002, FD003 and FD004 data in the C-MAPSS dataset. A considerable consistency is observed across the entire cycle range, signifying the LSTM’s capacity to maintain reliable predictions throughout various stages of engine operation. Discrepancies in certain sections are attributed to the increased prominence of fault signatures in the detection parameters when the RUL is low, emphasising the LSTM’s heightened sensitivity to impending engine degradation. Contrarily, in instances of high RUL, the LSTM exhibits enhanced accuracy, reflecting its proficiency in predicting the relatively healthy state of the engine. The overall RUL prediction accuracy overlaps at 98.916% with the actual RUL values through all FD001, FD002, FD003, FD004 data and establishes the effectiveness of the proposed method in delivering precise and reliable prognostications, substantiating its applicability in predictive maintenance scenarios.

## 4. Discussion

This study proposes a novel aircraft health monitoring solution based on predicting the RUL of turbofan engines. The study employs sensor data from 100 engines in the C-MAPSS dataset with 20,631 instances allocated for training and 13,096 for testing. By utilising sensor data in the C-MAPSS dataset, the study explores a classification strategy, labelling engines as “needs maintenance” or “healthy” based on RUL values. The LSTM architecture is crafted with a three-layer structure and demonstrates the innovative application of deep learning in the aviation domain. The study emphasises the benefits of the Adam optimisation algorithm, demonstrating its adaptability to the diverse scales and behaviours of LSTM parameters, overcoming challenges such as vanishing or exploding gradients. This adaptability is particularly crucial in capturing the intricate patterns of engine degradation dynamics, a characteristic not easily addressed by traditional methods. The piece-wise formulation of the RUL target function adds an extra layer of sophistication to the model, acknowledging the subtle degradation patterns inherent in engines. This unique approach aligns with the understanding that the degradation becomes noticeable only after a certain operational threshold, setting the foundation for a more context-aware predictive model. The study showcases the LSTM’s performance, achieving an accuracy of 98.916% and introduces the F1-score as a key metric in balancing precision and recall.

The obtained results highlight the effectiveness of LSTM in predicting the RUL of aircraft engines based on health monitoring data. The LSTM demonstrated accuracy with an overall precision of 93.88%, recall of 100% and an F1-score of 96.8%. This success is superior to traditional regression methods such as Gaussian Processes Regression and Random Forest Regression which demonstrated lower accuracy and precision. The high precision and recall values emphasise the LSTM’s capability to accurately identify engines in need of maintenance, crucial for proactive and cost-effective maintenance strategies. The LSTM’s robust performance across diverse engine cycles underscores its adaptability to varying operational conditions, providing reliable predictions throughout the entire RUL range. The attention mechanism incorporated into the LSTM architecture further enhances its ability to distinguish critical temporal patterns, making it particularly suitable for time-series analysis and sequential prediction tasks.

The importance of these discoveries is significant for predictive maintenance. The ability to accurately predict the RUL of critical components allows for optimised resource allocation, reduced downtime and improved safety. The novel approach of integrating an attention mechanism within the LSTM, combined with hyperparameter tuning through autotuning techniques, represents a methodological advancement. This approach not only underlines the importance of attention mechanisms in sequence modelling but also stresses the efficacy of autotuning for fine-tuning LSTM architectures, addressing challenges such as overfitting and adapting to dataset characteristics. The divergence from traditional encoder–decoder structures in applying attention mechanisms, coupled with automated hyperparameter tuning, differentiates this approach. The study contributes to the ongoing exploration of advanced machine learning techniques for prognostic tasks. The obtained results have been compared with traditional machine learning algorithms. It can be observed from Table 3 and Table 4 that LSTM achieves the highest performance for all metrics. A comparative analysis is presented in Table 5 presenting the outcomes of studies conducted on LSTM and its derivatives. The table provides a comprehensive view of the results obtained from these studies, offering insights into the performance metrics and achievements of LSTM variants. Each method is associated with its respective $P_{0,2}\left(P_{i}\right)$ , providing an understanding of their effectiveness.

The proposed LSTM, equipped with attention mechanisms and fine-tuned through autotuning techniques, emerges as a powerful and innovative instrument for predicting the RUL of aircraft engines. The high accuracy, precision and recall values emphasise its potential to revolutionise predictive maintenance practices, offering a more efficient and proactive approach to asset management in the industry.

## 5. Conclusions

This study has delved into the domain of advancing aircraft safety through the integration of LSTM into engine health monitoring systems. The study underscores the potential efficacy of LSTM in predicting and detecting engine anomalies, thereby contributing to the main goal of enhancing aviation safety. This research lays the foundation for continued exploration and innovation in critical aircraft safety, promoting a safer and more reliable aviation industry. LSTM has revealed a transformative approach to handling the intricate temporal dynamics of engine degradation. The significance of LSTM lies not only in its capacity to directly process raw time-series data but also in its ability to adapt dynamically to the evolving context of the dataset. The comprehensive exploration of LSTM architecture, from the GRU to the specialised structure of LSTM itself, has underscored the power of capturing past, present and future information crucial for predictive maintenance.

The integration of attention mechanisms with LSTM has proven to be a critical enhancement, allowing the LSTM to selectively focus on specific aspects of input sequences, thereby improving its ability to discern critical information. The novel approach of applying attention mechanisms independently from an encoder–decoder structure has demonstrated promising results. The application of attention mechanisms without an explicit encoder–decoder architecture offers several advantages:

1. Simplicity: The lack of dedicated encoder–decoder structure simplifies the model architecture, making it more straightforward and computationally efficient. This simplicity is advantageous in situations where a less complex model is desired when computational resources are limited.
2. Reduced Model Complexity: Encoder–decoder architectures are relatively complex, involving separate components for encoding and decoding. By avoiding this structure, the LSTM becomes more streamlined and requires fewer parameters, reducing the risk of overfitting and making it more interpretable.
3. Direct Interaction with Sequential Data: Direct interaction with hidden states from an LSTM is more intuitive and aligned with the nature of the problem. This direct interaction allows the attention mechanism to adapt dynamically to the evolving context within the sequence.
4. Improved Performance: Where the relationships between different time steps in the sequence are critical and straightforward, eliminating the encoder–decoder structure leads to improved performance. The direct use of hidden states in attention computation allows the LSTM to focus on relevant information throughout the sequence.
5. Reduced Inference Time: With a simplified architecture, inference time is reduced compared to more complex encoder–decoder designs. This is advantageous in real-time applications where rapid deployment is essential.

It is important to note that the choice of using an attention mechanism without an encoder–decoder structure depends on the nature of the task, the characteristics of the data and the specific goals of the modelling approach. On the other hand, Malhotra et al. have demonstrated the efficacy of the LSTM encoder-decoder-based restructuring model for detecting anomalies in time series data as evidenced by their studies [5,43]. Similarly, Gugulothu et al. [44] have asserted the suitability of the LSTM structure for predictive maintenance. The adoption of autotuning techniques for LSTM hyperparameter optimisation has further refined the LSTM’s performance, ensuring adaptability to the unique characteristics of the dataset and preventing issues like overfitting or underfitting. Autotuning the LSTM offers several advantages in the context of hyperparameter optimisation and model fine-tuning:

1. Efficient Hyperparameter Search: Autotuning automates the process of hyperparameter optimisation, efficiently exploring a wide range of hyperparameter configurations. This eliminates the need for manual tuning which is time-consuming and requires domain-specific expertise.
2. Improved Generalisation: Autotuning helps prevent overfitting by identifying hyperparameter settings that enhance the LSTM’s generalisation performance. It balances model complexity and avoids fitting the training data too closely, leading to better performance on unseen data.
3. Adaptability to Dataset Characteristics: LSTM performs differently based on the characteristics of the dataset. Autotuning allows the model to adapt to the specific properties of the data, optimising hyperparameters for tasks with varying input sequences and time dependencies.
4. Reduced Risk of Underfitting: Autotuning aids in the discovery of hyperparameters that prevent underfitting, ensuring that the LSTM is expressive enough to capture complex temporal dependencies and patterns within the data.
5. Increased Robustness Across Dataset: By identifying hyperparameters that generalise well, the LSTM becomes versatile and applicable to a broader range of scenarios.
6. Adaptive Learning Rates: Autotuning includes optimisation algorithms that adaptively adjust learning rates. Adaptive learning rates enhance the training process by dynamically modifying the step sizes for weight updates based on the gradients.

Autotuning in LSTM facilitates the hyperparameter optimisation process and directs the LSTM to generalise better to achieve improved performance without manual intervention. The efficiency, adaptability and time savings provided by autotuning contribute to the overall effectiveness of LSTM. The key advantage of the Adam algorithm is its adaptive learning rate which allows it to adjust the learning rates for each parameter individually based on their historical gradients. This adaptability makes the Adam algorithm well suited for training the LSTM. The Adam optimiser offers several benefits:

1. Adaptive Learning Rates: The Adam algorithm dynamically adapts the learning rates for each parameter based on their historical gradients. This adaptive learning rate mechanism helps overcome challenges associated with manually tuning learning rates, making it suitable for complex and dynamic datasets.
2. Efficient Memory Usage: The Adam algorithm maintains a moving average of both gradients and their squared values which results in more efficient memory usage compared to optimisations that require storing individual gradients for each parameter.
3. Combination of Momentum and RMSprop: The Adam algorithm combines the benefits of both momentum and RMSprop optimisation techniques. The momentum helps accelerate the optimisation process while the RMSprop adjusts the learning rates for each parameter individually.
4. Bias Correction: The Adam algorithm incorporates bias correction and this is especially beneficial in the early training stages when the estimates of the first and second moments are inaccurate. This correction helps stabilise and improve the optimisation process.
5. Effective Handling of Varying Gradient Information: The adaptive learning rates in the Adam algorithm make it resilient to varying gradient information. This adaptability allows the Adam algorithm to perform well across the dataset.

The Adam algorithm’s adaptive learning rate mechanism, along with its ability to handle sparse gradients and incorporate momentum, makes it a suitable choice for training complex architectures. It helps mitigate issues related to vanishing or exploding gradients, directing to more stable and efficient training processes. In conclusion, this study has proposed a practical and effective state-of-the-art solution in RUL prediction and introduced a novel approach to hyperparameter tuning and attention mechanism implementation. In light of the results, it is evident that the results obtained in this study align with the existing literature. When compared to previous studies, the results of this study stand out as being more successful.

## Figures and Tables

**Table 1:** Turbofan engine sensor description.

Number	Description	Units
1	Fan Inlet Temperature	^°^R
2	LPC Outlet Temperature	^°^R
3	HPC Outlet Temperature	^°^R
4	LPT Outlet Temperature	^°^R
5	Fan Inlet Pressure	psia
6	Bypass Duct Pressure	psia
7	HPC Outlet Pressure	psia
8	Physical Fan Speed	rpm
9	Physical Core Speed	rpm
10	Engine Pressure Ratio (P50/P2)	—
11	HPC Outlet Static Pressure	psia
12	Ratio of Fuel Flow to Ps30	pps/psia
13	Corrected Fan Speed	rpm
14	Corrected Core Speed	rpm
15	Bypass Ratio	—
16	Burner Fuel–Air Ratio	—
17	Bleed Enthalpy	—
18	Required Fan Speed	rpm
19	Required Fan Conversion Speed	rpm
20	High-Pressure Turbines Cool Airflow	lb/s
21	Low-Pressure Turbines Cool Airflow	lb/s

**Table 2:** LSTM results.

Metric	Result
MAE	1.284
Accuracy	98.916%
Precision	94.137%
Recall	100%
F1-score	97.33%

**Table 3:** Results for different regression methods.

Method	MAE	MSE	RAE	RSE	Accuracy
Linear Regression	20.72	28.581	0.577	0.487	74.45%
K-Nearest Neighbours Regression	21.29	29.678	0.593	0.516	76.37%
Support Vector Regression	23.33	29.991	0.646	0.547	78.13%
Gaussian Processes Regression	26.43	75.323	0.846	0.782	92.47%
Random Forest Regression	17.22	85.713	0.925	0.897	92.78%

**Table 4:** Results for Gaussian Processes Regression, Random Forest Regression and LSTM models.

Method	Gaussian Processes Regression	Random Forest Regression	LSTM
MAE	26.43	17.22	1.284
Accuracy	92.47%	92.78%	98.916%
Precision	79.98%	90.13%	93.88%
Recall	89.46%	89.97%	100%
F1-score	84.36%	90.19%	96.8%

**Table 5:** Comparison of the state-of-the-art solutions.

Studies	Method	$P_{0,2}\left(P_{i}\right)$
Wu et al. [40]	Vanilla LSTM	90
Yuan et al. [41]	LSTM	91.42
Zhao et al. [42]	Deep LSTM	91.7
Malhotra et al. [43]	Deep LSTM-ED	98.3
Ours	LSTM	98.783

## Data Availability

The data presented in this study are available at: https://web.archive.org/web/20190111164455/https://ti.arc.nasa.gov/m/project/prognostic-repository/CMAPSSData.zip (accessed on 23 May 2023).

## Abbreviations

The following abbreviations are used in this manuscript:

C-MAPSS	Commercial Modular Aero-Propulsion System Simulation
LSTM	Long Short-Term Memory
RUL	Remaining Useful Life
DSR	Dempster–Shafer Regression
SVM	Support Vector Machine
RNN	Recurrent Neural Network
MSE	Mean Squared Error
LSTM-ED	Encoder–Decoder Long Short-Term Memory
SBA	Similarity-Based Approach
MLP	Multi-Layer Perceptron
SVR	Support Vector Regression
RVR	Relevance Vector Regression
CNN	Convolutional Neural Network
GRU	Gated Recurrent Unit
DBN	Deep Belief Network
MODBNE	Multiobjective Deep Belief Network Ensemble
HPC	High-performance Computing
RMSE	Root Mean Square Error
MAE	Mean Absolute Error
MSE	Mean Squared Error
RAE	Relative Absolute Error
RSE	Relative Squared Error
